# Minimal Impact of COVID-19 Pandemic on the Mental Health and Wellbeing of People Living With Dementia: Analysis of Matched Longitudinal Data From the IDEAL Study

**DOI:** 10.3389/fpsyt.2022.849808

**Published:** 2022-03-09

**Authors:** Serena Sabatini, Holly Q. Bennett, Anthony Martyr, Rachel Collins, Laura D. Gamble, Fiona E. Matthews, Claire Pentecost, Eleanor Dawson, Anna Hunt, Sophie Parker, Louise Allan, Alistair Burns, Rachael Litherland, Catherine Quinn, Linda Clare

**Affiliations:** ^1^College of Medicine and Health, University of Exeter, Exeter, United Kingdom; ^2^Faculty of Medical Sciences, Population Health Sciences Institute, Newcastle University, Newcastle upon Tyne, United Kingdom; ^3^School of Social Sciences, Institute of Brain, Behaviour and Mental Health, University of Manchester, Manchester, United Kingdom; ^4^Innovations in Dementia Community Interest Company (CIC), Exeter, United Kingdom; ^5^Centre for Applied Dementia Studies, Bradford University, Bradford, United Kingdom; ^6^National Institute for Health Research (NIHR) Applied Research Collaboration South-West Peninsula, London, United Kingdom

**Keywords:** quality of life, wellbeing, sense of self, optimism, life orientation

## Abstract

**Objective:**

Research suggests a decline in the mental health and wellbeing of people with dementia (PwD) during the COVID-19 pandemic; however few studies have compared data collected pre-pandemic and during the pandemic. Moreover, none have compared this change with what would be expected due to dementia progression. We explored whether PwD experienced changes in mental health and wellbeing by comparing pre-pandemic and pandemic data, and drew comparisons with another group of PwD questioned on two occasions prior to the pandemic.

**Methods:**

Community-dwelling PwD enrolled in the IDEAL programme were split into two groups matched for age group, sex, dementia diagnosis, and time since diagnosis. Although each group was assessed twice, one was assessed prior to and during the pandemic (pandemic group; *n* = 115) whereas the other was assessed prior to the pandemic (pre-pandemic group; *n* = 230). PwD completed measures of mood, sense of self, wellbeing, optimism, quality of life, and life satisfaction.

**Results:**

Compared to the pre-pandemic group, the pandemic group were less likely to report mood problems, or be pessimistic, but more likely to become dissatisfied with their lives. There were no changes in continuity in sense of self, wellbeing, and quality of life.

**Discussion:**

Results suggest the pandemic had little effect on the mental health and wellbeing of PwD, with any changes observed likely to be consistent with expected rates of decline due to dementia. Although personal accounts attest to the challenges experienced, PwD appear to have been resilient to the impact of lockdown and social restrictions during the pandemic.

## Introduction

The COVID-19 pandemic (hereafter referred to as “the pandemic”) and consequent periods of lockdown and social restrictions forced individuals to change their routines, social interactions, and usual ways of accessing health and social care services (1–3). For instance, non-urgent clinical activities were postponed, conducted via phone, or interrupted. People considered clinically vulnerable were strongly advised to stay at home even in those periods when social restrictions were less severe. Clinically vulnerable people included some people with dementia (PwD) who also had other health conditions. Social support services for community dwelling PwD and their carers were also interrupted. PwD living in the community may have been particularly affected by these changes related to the pandemic.

However, existing evidence based on carer reports and self-reports from PwD provided mixed results (4, 5). On one hand some studies (6–10) found that the pandemic accelerated decline in the cognitive, physical, functional, and mental health of PwD, as well as increased neuropsychiatric symptoms, behavioral problems, and loneliness among PwD (3, 5–17). On the other hand, some studies found that the pandemic had minimal negative impact or no impact on these outcomes (7, 8, 12, 18). These inconsistent findings may be due to the methodological limitations of cross-sectional analyses based on self- and informant reports (6).

Although some studies examined changes in the mental health and wellbeing of PwD by using data collected both prior to and during the pandemic (3, 7, 11, 18, 19), they lacked a matched comparison group providing data on changes in mental health and wellbeing over time prior to the pandemic. Because of this they could not determine whether a decline in the mental health and wellbeing of PwD was due to the pandemic or to the normal progression of dementia. Studies such as that conducted by Tondo et al. (20) found that during the pandemic PwD experienced a greater cognitive decline compared to what was expected for people in their same stage of the illness. However, to the best of our knowledge, no study has undertaken a similar approach to that of Tondo et al. (20) to explore the effects of the pandemic on the mental health and wellbeing of PwD.

Using longitudinal data from the IDEAL (Improving the experience of Dementia and Enhancing Active Life) cohort, we found that during the pandemic PwD were more likely to report discontinuity in sense of self, poorer quality of life, and lower life satisfaction, but also better mood, greater optimism, and similar levels of physical health, when compared to mean scores for the cohort prior to the pandemic, but we could not establish whether the observed changes were attributable to conditions during the pandemic (19).

Building on our earlier findings and on longitudinal data from the IDEAL programme, the current study investigated the mental health and wellbeing of PwD by comparing longitudinal change seen in a group of participants assessed both prior to and during the pandemic with that seen in a matched comparison group of PwD assessed on two occasions prior to the pandemic. To capture mental health and wellbeing, measures assessing mood, continuity/discontinuity in sense of self, wellbeing, optimism, quality of life, and life satisfaction were employed. We hypothesized that, compared to the experience of their matched counterparts prior to the pandemic, PwD during the pandemic were more likely to be anxious or depressed, perceive discontinuity in sense of self, report lower psychological wellbeing, be less optimistic, have poorer quality of life, and be dissatisfied with their life. We also expected that the changes in mental health and wellbeing experienced by PwD during the pandemic would be greater than the changes over time experienced by a matched group of PwD whose data were collected during two assessment waves prior to the pandemic.

## Materials and Methods

### Design

We report a comparison of data from two matched groups of PwD, each assessed on two occasions. One group was assessed prior to and during the pandemic, and the other group was assessed on two occasions prior to the pandemic.

This study is embedded in the ongoing IDEAL programme. The IDEAL programme centers on a longitudinal cohort study following a large group of PwD and their carers for up to 6 years (21, 22). PwD were recruited for baseline interviews (T1) through 29 National Health Service (NHS) sites in England, Scotland, and Wales between 2014 and 2016. Participants lived in the community, and had a clinical diagnosis of dementia and a Mini-Mental State Examination (MMSE) (23) score ≥15 at baseline. For those who agreed to participate, trained researchers conducted structured interviews during home visits. Follow-up interviews occurred 12 (T2) and 24 (T3) months later. A follow-up study (IDEAL-2) began in 2018 and aimed to comprise three further waves of data collection (T4–T6); the T4 interviews were scheduled 2 years after T3. T4 was due to end in July 2020 and T5 was due to end 12 months later but data collection for both waves was interrupted by the COVID-19 pandemic. The INCLUDE (Identifying and mitigating the individual and dyadic impact of COVID-19 and life under physical distancing on people with dementia and carers) study was introduced at this point to understand the impact of COVID-19 on the IDEAL cohort. Those who had participated in IDEAL and IDEAL-2 were invited to take part in INCLUDE. Interviews for INCLUDE were conducted remotely by trained researchers between September 2020 and April 2021, see (24) for details.

### Participants

Two matched groups of participants were identified from the INCLUDE and IDEAL datasets:

- The “pandemic group” (*n* = 115) comprised PwD who were assessed for both IDEAL T3 and INCLUDE.
- The “pre-pandemic group” (*n* = 230) comprised PwD who were assessed for IDEAL T1 and T3 but did not take part in INCLUDE.

Participants in the pre-pandemic group were matched 2:1 to participants in the pandemic group. Matching was based on age group, sex, dementia diagnosis, and time since dementia diagnosis. The pool of participants from which the matched pre-pandemic group was chosen consisted of those who took part in IDEAL T1 and T3 but not INCLUDE (as described above, *n* = 736). Two participants from this pool were matched to each participant from the pandemic group. However, using these criteria it was not possible to identify two exact matches for every participant in the pandemic group. Therefore, for the first match, 70 out of 115 of the pandemic group were matched exactly on age group (<65, 65–69, 70–74, 75–79, 80+ years), sex, dementia subtype (Alzheimer's disease, vascular dementia, mixed Alzheimer's and vascular dementia, frontotemporal dementia, Parkinson's disease dementia, dementia with Lewy bodies, unspecified/other) and time since diagnosis (<1, 1–2, 3–5 6+ years). Another 14 out of 115 of the pandemic group were matched exactly on age group, sex, binary dementia subtype (Alzheimer's disease, vascular dementia, mixed, vs. other), and time since diagnosis. Seven out of 115 had exact first matches on age group, sex, binary dementia subtype, and binary time since diagnosis (≤ 2, ≥3 years). The remaining 24 from the pandemic group had exact first matches on age group, sex, and binary dementia diagnosis. For second matches 113 out of 115 matched on age group, sex, and binary dementia diagnosis, and two out of 115 matched only on age group, and sex.

There were two “waves” of data available for each group:

- Wave 1 (W1) refers to IDEAL T1 for the pre-pandemic group and IDEAL T3 for the pandemic group.
- Wave 2 (W2) refers to IDEAL T3 for the pre-pandemic group and INCLUDE data collection for the pandemic group.

These time-points were selected as the time between assessments was the nearest match we could achieve for the two groups.

### Measures

Single items from standardized measures were used to assess self-reported mood (depressed or anxious; not depressed or anxious) (25), continuity in sense of self (discontinuity; continuity) (24), wellbeing (high; moderate; low) (26), optimism (optimistic; pessimistic or neutral) (27), quality of life (poor or fair; good; excellent) (28), and life satisfaction (satisfied; dissatisfied) (29); see Supplementary Table 1.

Covariates included marital status (spouse/partner; single; widowed), education (no qualifications; school leaving certificate at age 16; school leaving certificate at age 18; university), social class (high; intermediate; low), living alone (yes; no), cognition [MMSE score (23) 0–21, 22–25, 26–30], diagnosed depression (depressed; not depressed) and months between waves. Number of health conditions other than dementia was a count of heart problems (heart attack or congestive heart failure), hypertension, peripheral vascular disease, stroke or hemiplegia, transient ischaemic attack, chronic bad chest, inflammation of the joints, peptic/stomach ulcer disease, skin ulcer, diabetes, moderate or severe kidney disease, cancer, and liver disease. The count was categorized into either 0–1 or 2+ co-morbidities.

### Statistical Methods

Binary outcomes (anxiety or depression, continuity in sense of self, optimism, life satisfaction) were analyzed using mixed effect logistic regression models with waves grouped within participant as a random intercept to account for correlation over waves within a participant. For outcomes with more than two categories (wellbeing, quality of life) mixed effect multinomial logistic regression models were used, again with the random intercept grouping waves within participants. Due to low participant numbers the random intercept was constrained to be equal for all outcome categories instead of having separate random intercepts for each category of the outcome. To understand the difference in trends over time (waves) in the outcome between the pre-pandemic group and the pandemic group, an interaction between the pandemic group indicator variable and wave variable was included in the model. All models were adjusted for the matching variables (age group, sex, dementia subtype, time since dementia diagnosis) and for other covariates that were important to the model. A sensitivity analysis was conducted for the mood model where diagnosed depression was included as a covariate to control for long-term depression.

## Results

In the whole sample (*n* = 345) the average age was 72.6 years and 48.7% were women. As participants were matched for age group rather than age, mean age was 72.7 years in the pre-pandemic group and 72.4 years in the pandemic group. In the whole sample the majority of participants had a partner, had education to age 18 or university level, were in the higher social class group, and had Alzheimer's disease (Table 1). Average time between waves was 24.9 months for the pre-pandemic group and 39.1 months for the pandemic group. Including time between waves as a covariate or interaction with pandemic group in the model did not lead to significantly different results, so this was excluded.

**Table 1:** Demographic profiles of the pre-pandemic and pandemic groups.

		**Pre-pandemic**		**Pandemic**	
		***N***	**%**	***N***	**%**
Age group	<65	38	16.5	19	16.5
	65–69	44	19.1	22	19.1
	70–74	56	24.4	28	24.4
	75–79	40	17.4	20	17.4
	≥80	52	22.6	26	22.6
		Mean:	72.7	Mean:	72.4
Sex	Men	118	51.3	59	51.3
	Women	112	48.7	56	48.7
Marital status	Spouse/partner	184	80.0	84	73.0
	Single	17	7.4	22	19.1
	Widowed	29	12.6	9	7.8
Education	No qualifications	64	27.8	23	20.4
	Qualification at 16	39	17.0	17	15.0
	Qualification at 18	78	33.9	49	43.4
	University	49	21.3	24	21.2
Social class	High	90	41.7	52	47.7
	Intermediate	96	44.4	36	33.0
	Low	30	13.9	21	19.3
Living situation	Living with others	191	83.4	91	79.1
	Living alone	38	16.6	24	20.9
Health condition count in addition to dementia	0–1	144	63.7	64	56.6
	2+	82	36.3	49	43.4
Diagnosed depression	Not depressed	189	83.6	93	82.3
	Depressed	37	16.4	20	17.7
Dementia subtype	Alzheimer's disease	142	61.7	61	53.0
	Vascular dementia	16	7.0	15	13.0
	Mixed (Alzheimer's and vascular)	38	16.5	21	18.3
	Frontotemporal dementia	13	5.7	8	7.0
	Parkinson's disease dementia	6	2.6	4	3.5
	Lewy body dementia	7	3.0	0	0.0
	Unspecified/Other	8	3.5	6	5.2
Length of time since	<1 year	93	42.5	0	0.0
Diagnosis	1–2 years	83	37.9	51	47.2
	3–5 years	37	16.9	43	39.8
	≥6 years	6	2.7	14	13.0
Average time (months) between W1 and W2		Mean	Range	Mean	Range
		24.9	18–38	39.1	27–51

Number and proportions of participants in each category of mental health and wellbeing indicators are reported in Table 2. Results from the mixed effect models are shown in Table 3.

**Table 2:** Outcomes at wave 1 and wave 2 in the pre-pandemic and pandemic groups.

		**Pre-pandemic**				**Pandemic**			
		**Wave 1**		**Wave 2**		**Wave 1**		**Wave 2**	
		***N***	**%**	***N***	**%**	***N***	**%**	***N***	**%**
Mood	Depressed or anxious	79	34.5	73	32.6	44	38.3	29	25.4
	Not depressed or anxious	150	65.5	151	67.4	71	61.7	85	74.6
Sense of self	Discontinuity	69	30.5	71	32.3	38	33.0	39	34.5
	Continuity	157	69.5	149	67.7	77	67.0	74	65.5
Wellbeing	Low	25	10.9	26	12.2	13	11.4	13	12.0
	Moderate	50	21.8	56	26.2	29	25.4	29	26.9
	High	154	67.3	132	61.7	72	63.2	66	61.1
Optimism	Pessimistic or neutral	59	26.1	70	32.0	37	32.2	29	25.7
	Optimistic	167	73.9	149	68.0	78	67.8	84	74.3
Quality of life	Poor or fair	37	16.2	51	23.1	25	21.7	31	27.4
	Good	128	56.1	117	52.9	69	60.0	54	47.8
	Excellent	63	27.6	53	24.0	21	18.3	28	24.8
Life satisfaction	Dissatisfied with life	36	16.1	33	15.0	10	8.7	16	14.2
	Satisfied with life	188	83.9	187	85.0	105	91.3	97	85.8

In the pre-pandemic group 34.5% at W1 and 32.6% at W2 were depressed or anxious. At W1 38.3% of the pandemic group were depressed or anxious, decreasing to 25.4% at W2; the trend for this decrease in feeling depressed or anxious in the pandemic group differed to the trend between W1 and W2 in the pre-pandemic group; interaction odds ratio (OR): 0.4, 95% confidence interval (CI): 0.1–1.0 (Table 3; Supplementary Figure 1). The results were robust when adjusted for diagnosed depression in the sensitivity analysis (Supplementary Table 2).

**Table 3:** Odds ratios from mixed effect logistic regression models and multinomial logistic regression models with 95% confidence intervals.

		**Pandemic vs. pre-pandemic at wave 1**		**Wave 2 vs. wave 1 for pre-pandemic group**		**Interaction between pandemic group and wave**	
		**OR**	**95% CI**	**OR**	**95% CI**	**OR**	**95% CI**
Mood^a^	Depressed or anxious	Ref.		Ref.		Ref.	
	Not depressed or anxious	1.1	0.5–2.8	0.8	0.5–1.4	0.4	0.1–1.0
Sense of sel^b^	Discontinuity	1.1	0.5–2.4	1.1	0.7–1.9	1.1	0.4–2.6
	Continuity	Ref.		Ref.		Ref.	
Wellbeing^c^	Low	1.1	0.4–3.1	1.2	0.6–2.4	1.0	0.3–3.4
	Moderate	1.0	0.4–2.4	1.5	0.8–2.6	0.8	0.3–2.1
	High	Ref.		Ref.		Ref.	
Optimism^d^	Pessimistic or neutral	1.4	0.7–2.8	1.5	0.9–2.4	0.5	0.2–1.2
	Optimistic	Ref.		Ref.		Ref.	
Quality of life^e^	Poor or fair	1.4	0.7–3.1	1.6	0.9–2.9	0.9	0.3–2.3
	Good	Ref.		Ref.		Ref.	
	Excellent	0.7	0.3–1.4	0.9	0.6–1.6	1.8	0.7–4.6

*Results for main effects and interaction between pandemic group indicator variable and wave variable. Main effect of wave gives the odds ratio (OR) comparing the pandemic groups to the pre-pandemic group at wave 1. Main effect for Wave compares wave 2 to wave 1 for the pre-pandemic group. The interaction compares the trend over waves in the pandemic group to the trend over waves in the pre-pandemic group. 95% confidence interval (95% CI); Ref. indicates reference category for the outcome. All models adjusted for age group, sex, binary time since diagnosis, and binary dementia diagnosis*.

a *Additionally adjusted for education, health condition count, and MMSE group*.

b *Additionally adjusted for education, marital status, health condition count, depression diagnosis, and MMSE group*.

c *Additionally adjusted for social class, and marital status*.

d *Additionally adjusted for education, marital status, health condition count, and depression diagnosis*.

e *Additionally adjusted for education, social class, marital status, health condition count, depression diagnosis, and MMSE group*.

In the pre-pandemic group (W1: 30.5%, W2: 32.3%) and pandemic group (W1: 33.0%, W2: 34.5%) there was no evidence of change in the proportion of participants reporting discontinuity in sense of self and no evidence of any difference in trends between groups (interaction OR: 1.1, 95% CI: 0.4–2.6) (Table 3; Supplementary Figure 2).

At W1 67.3% of the pre-pandemic group had a high level of wellbeing; by W2 this had dropped to 61.7%. In the pandemic group the proportion of participants reporting high levels of wellbeing was similar at W1 (63.2%) and W2 (61.1%). There was some evidence to suggest an increase in the proportion reporting moderate wellbeing by W2 in the pre-pandemic group (OR: 1.5, 95% CI: 0.8–2.6) with no difference in trend for the pandemic group (interaction OR: 0.8, 95% CI: 0.3–2.1, Table 3).

Most (73.9%) of the pre-pandemic group were optimistic at W1, reducing to 68.0% at W2. At W1 67.8% of the pandemic group were optimistic, increasing to 74.3% at W2 (Supplementary Figure 3). The models provided some evidence to suggest a difference in trends between the two groups (interaction OR: 0.5, 95% CI: 0.2–1.2) (Table 3; Supplementary Figure 3).

The proportion of those reporting good quality of life decreased for the pre-pandemic group (W1: 56.1%, W2: 52.9%) and pandemic group (W1: 60.0%, W2: 47.8%). Whereas, in the pre-pandemic group there was some evidence to suggest an increase in the proportion feeling their quality of life was poor or fair (OR: 1.6, 95% CI: 0.9–2.9), in the pandemic group there was some evidence to suggest an increase in the proportion feeling quality of life was excellent (interaction OR: 1.8, 95% CI: 0.7–4.6), as well as in the proportion feeling quality of life was poor or fair (Table 3).

The proportion reporting they were satisfied with life in the pre-pandemic group was similar at W1 (83.9%) and W2 (85.0%). At W1, 91.3% of the pandemic group were satisfied with life, decreasing to 85.8% at W2. There was some evidence to show the trend over waves differed between the pre-pandemic and pandemic group (interaction OR: 3.3, 95% CI: 0.9–13.0, Supplementary Table 3). However, these results should be interpreted with caution as, due to low numbers of those dissatisfied with life, the estimate for the interaction was inflated when adjusting for matching variables. Adjustment for further covariates did not improve the estimates (Supplementary Table 3).

## Discussion

This study investigated whether the experience of living through the pandemic and associated social restrictions affected the mental health and wellbeing of PwD living in the community in Britain. Contrary to our hypotheses, COVID-19 restrictions appeared to have little negative impact on whether PwD experienced continuity in sense of self and on how PwD appraised their wellbeing and quality of life, and in the case of mood and optimism, COVID-19 restrictions appeared to have a positive impact. Although the hypothesis that, compared to pre-COVID-19, PwD during COVID-19 were more likely to be dissatisfied with their lives was partially supported, estimations were inflated. Overall, findings for six outcomes capturing different facets of mental health and wellbeing consistently suggest that, when considering PwD enrolled in the IDEAL cohort as a group, the pandemic was associated with minimal negative change in mental health and wellbeing and with an improvement in mood and optimism.

The small decline in the proportion of PwD who reported mood problems in both the pre-pandemic and pandemic groups is consistent with previous evidence (19, 30). This effect was larger in the pandemic group, suggesting that some circumstances related to COVID-19 led to a reduction in the proportion of PwD having mood problems. It may be that during lockdown carers provided greater social support to PwD, or, as qualitative studies have found, PwD perceived the home environment as a safe place where they could enjoy quiet time, learn new skills, or return to past hobbies without fearing failure or comparison with peers (31). The engagement of PwD in activities such as reading and playing computer games during the lockdown is documented in other studies (7, 19). Due to stigma, embarrassment or awareness of being less able to engage in activities in normal times PwD may experience social anxiety and, as a consequence, withdraw from social activities (32–36), so in this sense restrictions may have had positive aspects. Nonetheless, further understanding of the mechanisms through which pandemic experiences decreased the likelihood of mood problems in PwD could help to identify ways of promoting better mood in PwD post-pandemic.

This was the first study exploring whether the pandemic had an influence on continuity in sense of self in PwD. We found that COVID-19 restrictions had no impact on continuity in sense of self in PwD. Again, it may be that during the lockdown PwD engaged in a range of activities and hobbies that contributed to the experience of continuity in sense of self.

Between September 2020 and April 2021, the pandemic did not influence the wellbeing and quality of life of PwD and only marginally influenced their optimism. Indeed, although PwD in the pandemic group were more likely to be optimistic about the future compared to those in the pre-pandemic group, differences between groups were minimal. Moreover, PwD who were more optimistic about the future prior to the pandemic maintained this optimistic outlook during the pandemic. This pattern of results suggests that the pandemic may not have influenced pre-existing levels of optimism (O'Rourke, 2015). This may be due to optimism being a relatively stable trait among older people (37).

There was some evidence to support the hypothesis in relation to life satisfaction, as during the pandemic PwD were more likely to feel dissatisfied with their lives. However, the number of PwD expressing dissatisfaction with their lives was low both before and during the pandemic, and this significant effect is due to only six people becoming dissatisfied with their lives during COVID-19. Comparison of baseline levels of life satisfaction between the pandemic group and the pre-pandemic group also shows that the proportion of PwD feeling dissatisfied with their lives was lower in the pandemic group and, despite a significant increase in the number of PwD becoming dissatisfied with their life during COVID-19, this number remained lower than the pre-pandemic group.

Compared to previous evidence (5) this study provides a more positive picture of the mental health and wellbeing of PwD during COVID-19. The disparity with previous studies on this topic may be due to several major methodological differences. First, although previous longitudinal studies identified a decline in the mental health and wellbeing of PwD during the pandemic compared with pre-pandemic information (11, 38–42), they could not discern whether the observed change was due to the typical course of dementia or to the impact of pandemic-related social restrictions. In contrast, by using longitudinal data to compare the change experienced by PwD during the pandemic with that experienced by a matched sample of PwD whose data were collected prior to COVID-19, our results suggest that most of the negative changes experienced by PwD during the pandemic are parallel to those that would be expected in a group of PwD with similar demographic characteristics under normal conditions. For instance, although the current analyses confirmed some of the previous results from the IDEAL cohort (e.g., a decrease in mood problems) that were based on comparison of pre-pandemic and pandemic data without a matched comparison group (19) it did not confirm others (e.g., poorer quality of life). The methodological advancements of this study suggest that the previously-described increase in the proportion of PwD reporting discontinuity in sense of self, poorer quality of life, and dissatisfaction with their life may have been due to the progress of dementia rather than to the pandemic.

Second, another possible reason for the disparity between our findings and those of previous studies is that previous studies relied on informant ratings provided by carers (7, 10, 12, 17, 43–45) whereas this study considered the self-ratings of PwD. As carer ratings differ from self-ratings (46) and the subjective perceptions of people with mild-to-moderate dementia are widely accepted as valid (47), our study may have produced more reliable results than existing research. Third, whereas many previous studies collected data at the beginning of the pandemic and therefore during the period of strict lockdown (5), data collection for this study started in September 2020 and therefore encompassed both periods of lockdown and periods of significant easing of restrictions. The more positive results found in this study may be due to participants having had the time to overcome the initial shock caused by the pandemic, adapt to a new lifestyle, and cope with changes related to the pandemic and/or to people looking forward to enjoy reduced restrictions (48).

Fourth, as those IDEAL participants who could not use telephones and/or had deteriorated markedly since their previous assessment were underrepresented in INCLUDE, study analyses are based on a self-selected group of PwD who may have been well-positioned to adapt to the lockdown and social restrictions. Hence, our positive results may not generalize to all PwD. Fifth, many individual effects of the pandemic on the mental health and wellbeing of PwD may have remained hidden in our analyses that considered PwD as a group. Indeed, qualitative studies suggest a heterogenous picture in relation to the mental health and wellbeing of PwD during the pandemic (48). For instance, the majority of our sample lived with someone else. However, those PwD who live alone found it harder to cope with some of the changes related to the pandemic, such as increased loneliness, and were therefore more likely to experience poorer mental health and wellbeing (48).

Overall, study results suggest that COVID-19 did not have a negative impact on the mental health and wellbeing of our sample of PwD. Instead, it may have had a small beneficial effect particularly in relation to mood. Qualitative interviews conducted during the pandemic with 11 PwD participating in the IDEAL study and their carers suggest that in some cases the resilience shown by PwD may reflect positive social circumstances and use of previously-learned coping strategies (48). Despite our positive pattern of results, we should be mindful that some negative feelings and experiences, such as the perception of being abandoned by services, have been reported by PwD (19, 48). These should be addressed as they may lead to poorer mental health and wellbeing in the long term.

This study has some limitations. Exact matches on all variables could not be found for everyone in the pandemic group. However, all matching variables were included as covariates in the models, and in an effort to mitigate this limitation, the pre-pandemic group included two matches for each PwD in the pandemic group. Mental health and wellbeing were assessed with single-item measures that, although reducing the burden for participants (19), may not have adequately captured the constructs. There was also a longer timeframe between assessments for the pandemic group. However, the models controlling for the difference between timepoints led to the same results as those models not controlling for this difference between timepoints, suggesting that the difference between timepoints did not influence study findings. Moreover, there was generally little effect from COVID-19 on the mental health and wellbeing of PwD so it was unlikely that the longer gap between assessments for the pandemic group affected the results, especially as the expectation was for a greater effect from COVID-19 on this group.

Despite these limitations, our unique study design made it possible to investigate whether observed changes in mental health and wellbeing were due to the pandemic and concomitant social restrictions rather than reflecting the typical course for PwD. In this sample of PwD the COVID-19 pandemic appears to have had little to no negative effect on mental health and wellbeing, and little impact on continuity and discontinuity in sense of self, wellbeing, quality of life, and life satisfaction. If anything, there was a small positive impact on mood and optimism. Future research is needed to understand the mechanisms behind this unexpected positive effect as it may help to identify ways to address mood disturbance in PwD. The remarkable resilience shown by PwD in this study adds to current understanding of what is possible in adjusting to the diagnosis and living with the condition and offers hope that it is possible to “live well” with dementia. Although this study found that the radical changes imposed by the pandemic did not have a negative effect on the mental health and wellbeing of PwD as a group, some PwD may have been less resilient than others. Indeed, previous studies documented how some PwD had negative experiences of the pandemic (19, 48). Future research could therefore focus on identifying the characteristics of those PwD who found it harder to adapt to the pandemic and would therefore benefit the most from post-pandemic support. Finally, as the social support provided by carers may be one of the reasons why PwD showed resilience in being able to maintain their mental health and wellbeing against the radical changes imposed by the pandemic, future work with the IDEAL dataset will explore whether carers' mental health and wellbeing have instead been affected during the pandemic.

## Data Availability Statement

The datasets presented in this article are not readily available because IDEAL data were deposited with the UK data archive in April 2020 and will be available to access from April 2023. Details of how the data can be accessed after that date can be found at: http://reshare.ukdataservice.ac.uk/854293/. INCLUDE data will be archived in connection with the IDEAL dataset in March 2022. Requests to access the datasets should be directed to http://reshare.ukdataservice.ac.uk/854293/ or Linda Clare L.Clare@exeter.ac.uk.

## Ethics Statement

INCLUDE was approved by Wales Research Ethics Committee 5 as an amendment to IDEAL-2 for England and Wales (18/WS/0111 AM12). IDEAL was approved by Wales Research Ethics Committee 5 (reference 13/WA/0405) and IDEAL-2 by Wales Research Ethics Committee 5 (reference 18/WS/0111) and Scotland A Research Ethics Committee (reference 18/SS/0037). IDEAL and IDEAL-2 are registered with the UK Clinical Research Network (UKCRN), numbers 16593 and 37955, respectively. The patients/participants provided their written informed consent to participate in this study.

## Author Contributions

LC, AM, CP, RC, LA, AB, RL, CQ, FM, SS, and HB conception and design. HB conducted study analysis and drafted the methods and results sections. SS interpreted the data and drafted the introduction and discussion sections. All authors critical appraisal and review of the manuscript and final approval of the manuscript.

## Funding

Identifying and mitigating the individual and dyadic impact of COVID-19 and life under physical distancing on people with dementia and carers (INCLUDE) was funded by the Economic and Social Research Council (ESRC) through grant ES/V004964/1. Investigators: LC, Victor, C., FM, CQ, Hillman, A., AB, LA, RA, AM, RC, and CP. ESRC is part of UK Research and Innovation (UKRI). Improving the experience of Dementia and Enhancing Active Life: living well with dementia. The IDEAL study was funded jointly by the Economic and Social Research Council (ESRC) and the National Institute for Health Research (NIHR) through grant ES/L001853/2. Investigators: LC, I. R. Jones, C. Victor, J. V. Hindle, R. W. Jones, M. Knapp, M. Kopelman, RL, A. Martyr, FM, R. G. Morris, S. M. Nelis, J. A. Pickett, CQ, J. Rusted, and J. Thom. IDEAL data were deposited with the UK data archive in April 2020 and will be available to access from April 2023. Details of how the data can be accessed after that date can be found at: http://reshare.ukdataservice.ac.uk/854293/. Improving the experience of Dementia and Enhancing Active Life: a longitudinal perspective on living well with dementia. The IDEAL-2 study was funded by Alzheimer's Society, grant number 348, AS-PR2-16-001. Investigators: LC, I. R. Jones, C. Victor, C. Ballard, A. Hillman, J. V. Hindle, J. Hughes, R. W. Jones, M. Knapp, RL, AM, FM, R. G. Morris, S. M. Nelis, CQ, and J. Rusted.

## Author Disclaimer

The views expressed are those of the author(s) and not necessarily those of the ESRC, UKRI, NIHR, the Department of Health and Social Care, the National Health Service, or Alzheimer's Society. The support of ESRC, NIHR and Alzheimer's Society is gratefully acknowledged.

## Conflict of Interest

RL was employed by Innovations in Dementia CIC. The remaining authors declare that the research was conducted in the absence of any commercial or financial relationships that could be construed as a potential conflict of interest.

## Publisher's Note

All claims expressed in this article are solely those of the authors and do not necessarily represent those of their affiliated organizations, or those of the publisher, the editors and the reviewers. Any product that may be evaluated in this article, or claim that may be made by its manufacturer, is not guaranteed or endorsed by the publisher.
