# Supplementary material for: Minimal Impact of COVID-19 Pandemic on the Mental Health and Wellbeing of People Living With Dementia: Analysis of Matched Longitudinal Data From the IDEAL Study
Source: Front Psychiatry. 2022 Mar 9;13:849808. doi: 10.3389/fpsyt.2022.849808 (PMC8965515; doi:10.3389/fpsyt.2022.849808)
Supplement: Supplementary file 1 [file Data_Sheet_1.docx]

**Minimal impact of COVID-19 on the mental health and well-being of people living with dementia: analysis on matched longitudinal data from the IDEAL study**

**Supplementary Table 1: Questions and response options for health and well-being outcomes.**

| **Outcome** | **Question** | **Response options** | **Analysis groupings** |
| --- | --- | --- | --- |
| Anxiety or depression (The EuroQol Group, 1990) | Do you feel anxious or depressed today? | I am not anxious or depressed, I am moderately anxious or depressed, I am extremely anxious or depressed | Depressed or anxious: I am moderately anxious or depressed, I am extremely anxious or depressed |
|  |  |  | Not depressed or anxious: I am not anxious or depressed |
| Sense of self (Clare et al., 2020) | I am still the same person as I have always been | Strongly disagree, disagree, neutral, agree, strongly agree | Discontinuity: Strongly disagree, disagree, neutral |
|  |  |  | Continuity: agree, strongly agree |
| Optimism (Scheier et al., 1994) | Overall, I expect more good things to happen to me than bad | Strongly disagree, disagree, neutral, agree, strongly agree | Optimistic: agree, strongly agree |
|  |  |  | Pessimistic or neutral: neutral, disagree, strongly disagree |
| Life satisfaction (Diener et al., 1985) | Are you basically satisfied with your life | Yes, no | Satisfied with life: Yes |
|  |  |  | Dissatisfied with life: No |
| Quality of life (Logsdon et al., 2000) | How would you describe your life as a whole? | Poor, fair, good, excellent | Poor or fair |
|  |  |  | Good |
|  |  |  | Excellent |
| Well-being (Bech, 2004) | In the last two weeks, how much of the time have you felt cheerful and in good spirits? | At no time, some of the time, less than half of the time, more than half of the time, most of the time, all of the time | High: most of the time, all of the time |
|  |  |  | Moderate: less than half of the time, more than half of the time |
|  |  |  | Low: at no time, some of the time |

**Supplementary Table 2: Sensitivity analysis for anxiety or depression including diagnosed depression as a covariate. Odds ratios from mixed effect logistic regression model of anxiety or depression with 95% confidence intervals.**

|  |  | **Pandemic vs. pre-pandemic group at Wave 1** | | **Wave 2 vs. Wave 1 for pre-pandemic group** | | **Interaction between pandemic group and Wave** | |
| --- | --- | --- | --- | --- | --- | --- | --- |
|  |  | **OR** | **95% CI** | **OR** | **95% CI** | **OR** | **95% CI** |
| Mood^1^ | Depressed or anxious | Ref. |  | Ref. |  | Ref. |  |
| (Main analysis) | Not depressed or anxious | 1.1 | 0.5 – 2.8 | 0.8 | 0.5 – 1.4 | 0.4 | 0.1 – 1.0 |
| Mood^2^ | Depressed or anxious | Ref. |  | Ref. |  | Ref. |  |
| (Sensitivity analysis) | Not depressed or anxious | 1.1 | 0.4 – 2.6 | 0.8 | 0.5 – 1.4 | 0.4 | 0.1 – 1.0 |

Note: odds ratio (OR); 95% confidence interval (95% CI); Ref. indicates the reference category used for the outcome.

^1^Main analysis, same results as in Table 2. Adjusted for age group, sex, binary time since diagnosis and binary dementia diagnosis, education, health condition count, and MMSE group

^2^Sensitivity analysis includes all covariates from (1) plus diagnosed depression

**Supplementary Table 3: Odds ratios from mixed effect logistic regression models of life satisfaction with 95% confidence intervals.**

|  |  | **Pandemic vs. pre-pandemic at Wave 1** | | **Wave 2 vs. Wave 1 for pre-pandemic group** | | **Interaction between pandemic group and Wave** | |
| --- | --- | --- | --- | --- | --- | --- | --- |
|  |  | **OR** | **95% CI** | **OR** | **95% CI** | **OR** | **95% CI** |
| Life Satisfaction^1^ | Satisfied with life | Ref. |  | Ref. |  | Ref. |  |
| (matching covariates) | Dissatisfied with life | 0.3 | 0.1 – 1.1 | 0.9 | 0.5 – 1.8 | 3.3 | 0.9 – 13.0 |
| Life satisfaction^2^ | Satisfied with life | Ref. |  | Ref. |  | Ref. |  |
| (other covariates) | Dissatisfied with life | 0.3 | 0.1 – 1.0 | 0.9 | 0.4 – 1.7 | 3.7 | 0.9 – 15.2 |

Note: Abbreviations include odds ratio (OR), 95% confidence interval (95% CI) and Ref. indicates the reference category used for the outcome.

^1^Adjusted for age group, sex, binary time since diagnosis, and binary dementia diagnosis

^2^Adjusted for age group, sex, binary time since diagnosis, and binary dementia diagnosis, education, social class, marital status, and health condition count

**Supplementary Figure 1: Predicted probability of anxiety or depression at Wave one and Wave two for people with dementia in the pre-pandemic and pandemic groups**

**
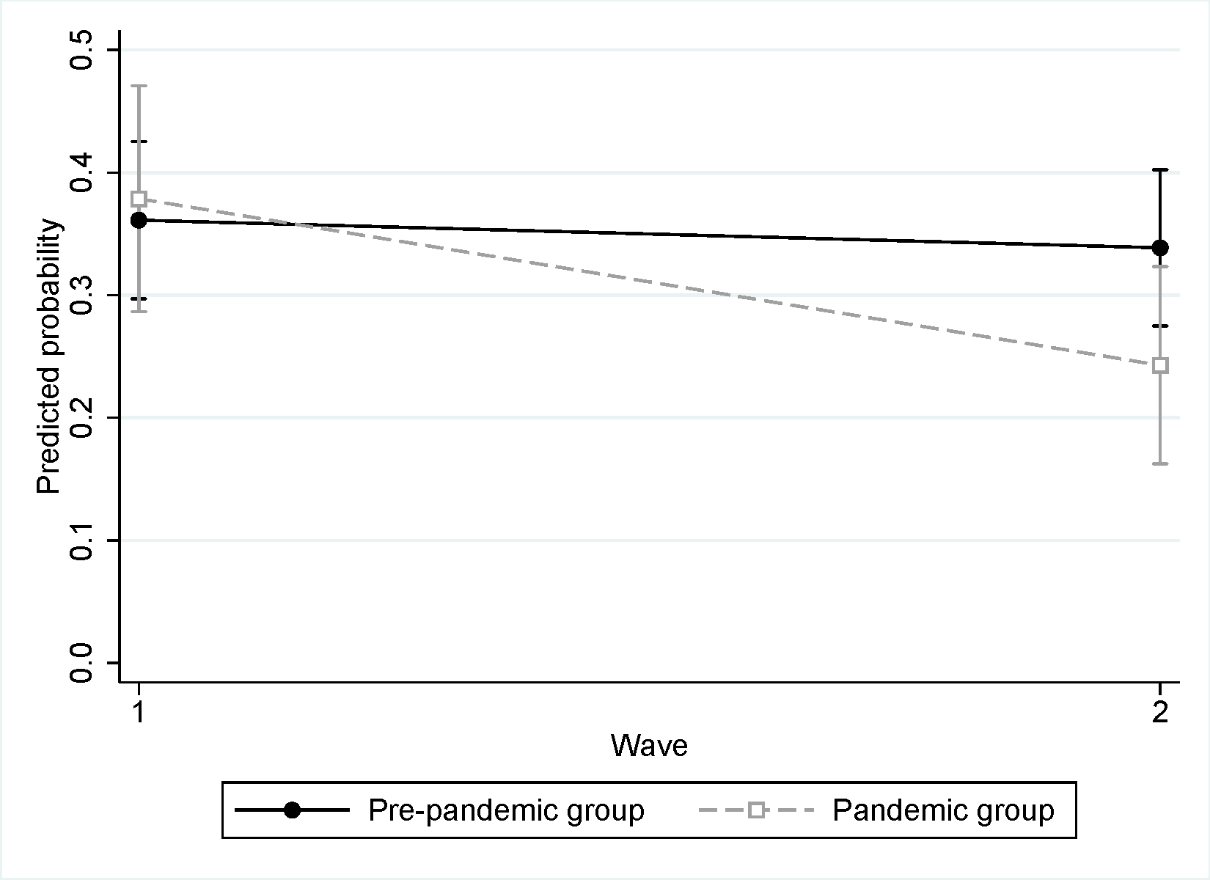
**

**Supplementary Figure 2: Predicted probability of experiencing discontinuity in sense of self at Wave one and Wave two for people with dementia in the pre-pandemic and pandemic groups.**

**
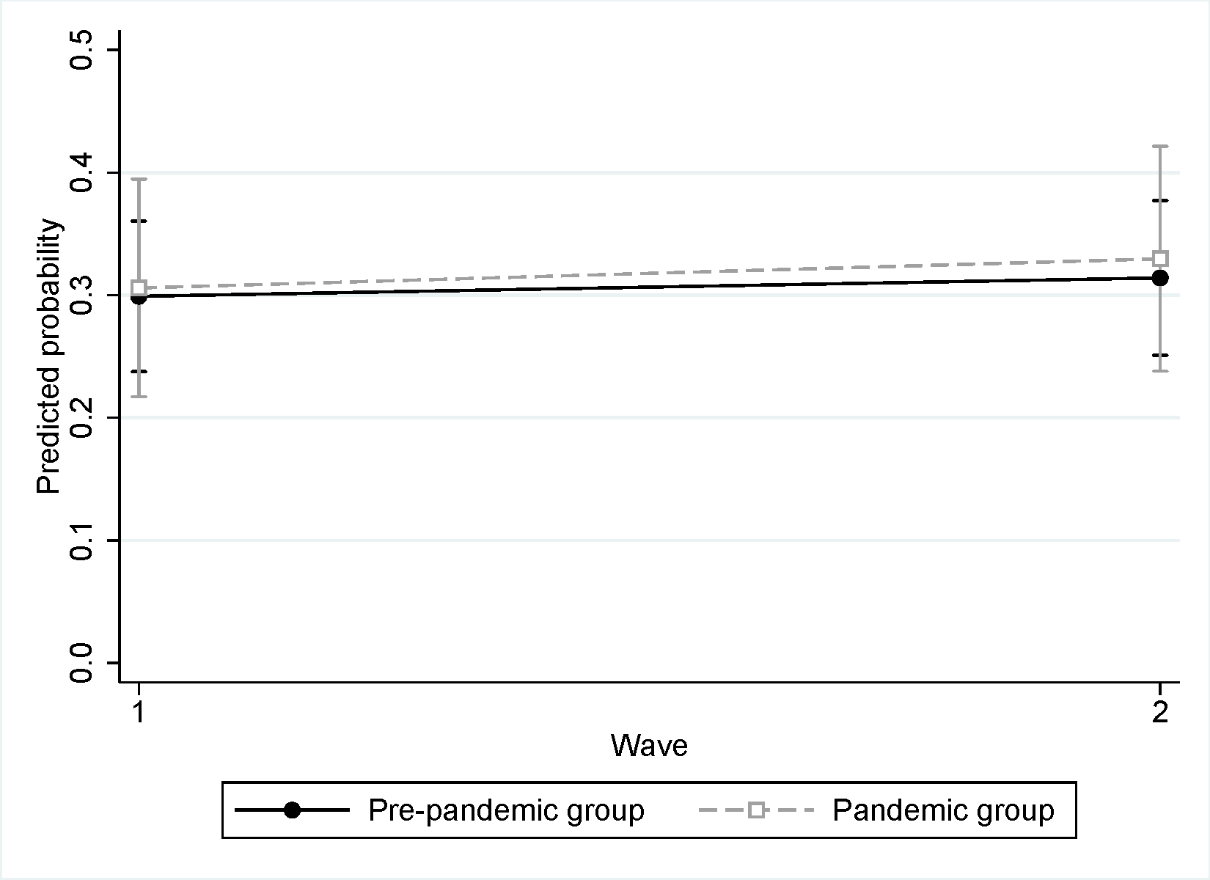
**

**Supplementary Figure 3: Predicted probability of feeling optimistic at Wave one and Wave two for people with dementia in the pre-pandemic and pandemic groups.**

**
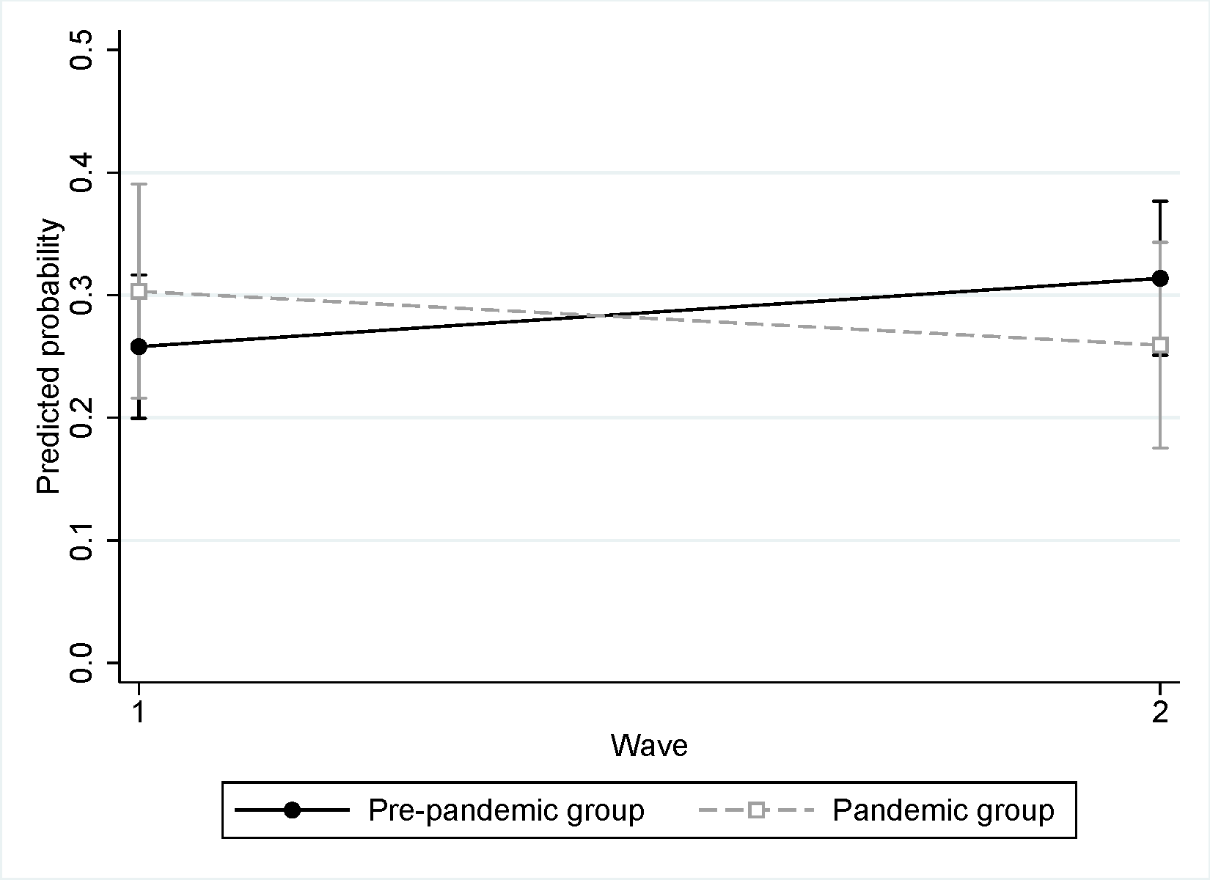
**

**References**

Bech, P. (2004). Measuring the dimension of psychological general well-being by the WHO-5. *Quality of Life Newsletter* 32**,** 15-16.

Clare, L., Martyr, A., Morris, R.G., and Tippett, L.J. (2020). Discontinuity in the subjective experience of self among people with mild-to-moderate dementia is associated with poorer psychological health: findings from the IDEAL cohort. *Journal of Alzheimer's Disease* 77**,** 127-138.

Diener, E., Emmons, R.A., Larsen, R.J., and Griffin, S. (1985). The Satisfaction With Life Scale. *Journal of Personality Assessment* 49**,** 71-75.

Logsdon, R.G., Gibbons, L.E., Mccurry, S.M., and Teri, L. (2000). "Quality of life in Alzheimer's disease: patient and caregiver reports," in *Assessing quality of life in dementia,* eds. S.M. Albert & R.G. Logsdon. (New York: Springer), 17-30.

Scheier, M.F., Carver, C.S., and Bridges, M.W. (1994). Distinguishing optimism from neuroticism (and trait anxiety, self-mastery, and self-esteem): a reevaluation of the Life Orientation Test. *Journal of Personality and Social Psychology* 67**,** 1063-1078.

The Euroqol Group (1990). EuroQol - a new facility for the measurement of health-related quality of life. *Health Policy* 16**,** 199-208.
